# High-risk chest radiographic features associated with COVID-19 disease severity

**DOI:** 10.1371/journal.pone.0245518

**Published:** 2021-01-14

**Authors:** Sean Wei Xiang Ong, Terrence Chi Hong Hui, Yeong Shyan Lee, Salahudeen Mohamed Haja Mohideen, Barnaby Edward Young, Cher Heng Tan, David Chien Lye

**Affiliations:** 1 National Centre for Infectious Diseases, Singapore, Singapore; 2 Department of Infectious Diseases, Tan Tock Seng Hospital, Singapore, Singapore; 3 Department of Diagnostic Radiology, Tan Tock Seng Hospital, Singapore, Singapore; 4 Department of Diagnostic Radiology, Singapore General Hospital, Singapore, Singapore; 5 Lee Kong Chian School of Medicine, Nanyang Technological University, Singapore, Singapore; 6 Yong Loo Lin School of Medicine, National University of Singapore, Singapore, Singapore; Faculty of Science, Ain Shams University (ASU), EGYPT

## Abstract

**Objectives:**

High-risk CXR features in COVID-19 are not clearly defined. We aimed to identify CXR features that correlate with severe COVID-19.

**Methods:**

All confirmed COVID-19 patients admitted within the study period were screened. Those with suboptimal baseline CXR were excluded. CXRs were reviewed by three independent radiologists and opacities recorded according to zones and laterality. The primary endpoint was defined as hypoxia requiring supplemental oxygen, and CXR features were assessed for association with this endpoint to identify high-risk features. These features were then used to define criteria for a high-risk CXR, and clinical features and outcomes of patients with and without baseline high-risk CXR were compared using logistic regression analysis.

**Results:**

109 patients were included. In the initial analysis of 40 patients (36.7%) with abnormal baseline CXR, presence of bilateral opacities, multifocal opacities, or any upper or middle zone opacity were associated with supplemental oxygen requirement. Of the entire cohort, 29 patients (26.6%) had a baseline CXR with at least one of these features. Having a high-risk baseline CXR was significantly associated with requiring supplemental oxygen in univariate (odds ratio 14.0, 95% confidence interval 3.90–55.60) and multivariate (adjusted odds ratio 8.38, 95% CI 2.43–28.97, *P* = 0.001) analyses.

**Conclusion:**

We identified several high-risk CXR features that are significantly associated with severe illness. The association of upper or middle zone opacities with severe illness has not been previously emphasized. Recognition of these specific high-risk CXR features is important to prioritize limited healthcare resources for sicker patients.

## Introduction

Although chest radiography (CXR) is less sensitive compared to computed tomography (CT) in assessment of lung pathology, it is an attractive triage tool given its cost, portability, and ease of decontamination. While CT radiographic features of COVID-19 have been extensively described in the literature [1, 2], describing an interstitial pneumonitis with typical bilateral lower zone ground-glass opacities, there are fewer studies reporting CXR features [3]. CT has been used as the primary imaging modality in China where the outbreak first originated, however, caution has been advised regarding the overuse of CT for diagnosis of COVID-19, especially in areas with low incidence and hence lower pre-test probability [4]. CXR has been used extensively elsewhere in the initial assessment of COVID-19 patients, yet the exact role of CXR in triage and monitoring remains unclear [5], and there may be limitations as a normal CXR has been reported to be prevalent in COVID-19, with radiographic changes only subsequently picked up on CT [6].

Several quantitative CT radiographic scores have been developed to predict patients at risk of developing severe illness [7, 8]. Recently, our group showed that an objective CXR radiographic score, modified from the Radiographic Assessment of Lung Edema (RALE) score for acute respiratory distress syndrome (ARDS), could quantify disease severity associated with supplemental oxygen requirement and mechanical ventilation, and could predict clinical deterioration if done within six to ten days from illness onset [9]. Borghesi et al have also demonstrated the value of CXR in assessing disease severity using a similar scoring system [10]. However, such quantitative scores require time and radiologist expertise, limiting wider implementation especially in settings without access to prompt reporting by radiologists.

We aimed to identify qualitative high-risk CXR features in COVID-19 patients that can be easily assessed in the clinical setting, and which correlate with severe COVID-19 requiring oxygen (Stage IIB of the pulmonary phase as proposed by Siddiqi et al [11]), and define specific high-risk CXR criteria for routine clinical use.

## Materials and methods

We conducted a retrospective cohort study of all COVID-19 patients with baseline CXR admitted to our center between January 22 and March 15 2020, as part of a separate study that developed a quantitative COVID-19 radiographic score [9]. During this study period, all patients with confirmed COVID-19 infection were admitted for inpatient care and isolation, regardless of disease severity. Inclusion criterion was confirmed COVID-19 infection defined by positive SARS-CoV-2 specific real-time reverse transcriptase polymerase chain reaction on nasopharyngeal swab. Exclusion criteria were suboptimal baseline CXR limiting adequate interpretation, missing clinical data, or not having an CXR taken.

Clinical data were collected from the medical record by study investigators using an anonymized case report form adapted from the International Severe Acute Respiratory and Emerging Infection Consortium (ISARIC) [12], and data stored on a secured server. Data were collected until day 21 of admission or discharge. The primary endpoint was defined as requiring supplemental oxygen for hypoxia (oxygen saturation ≤94%). Informed consent was waived as there was no direct interaction with the patients. Clinical data were collected as part of a separate retrospective cohort study of COVID-19 patients approved by the Institutional Review Board (National Healthcare Group Domain Specific Review Board reference number 2020/01122).

The digital radiography units used were FDR Visionary UD 150L-40 flat-panel detector system and FDR Nano portable compact digital X-ray cart with flat panel sensor DR-ID 1201SE (Fujifilm, Tokyo, Japan). CXRs were acquired in either erect posteroanterior (PA) or sitting/supine anteroposterior (AP) projections depending on the patient’s ambulatory status. Images were exported in an anonymized format and reviewed on 2048x2048-pixel DICOM monitors (Barco, Sunnyvale, CA, USA) by three independent fully-accredited radiologists who were blinded to the patients’ clinical status or diagnosis. Presence of pulmonary opacities were recorded according to zones (upper, middle, and lower) and laterality (right and left) after consensus between the three radiologists. To simplify interpretation, no distinction was made between different pulmonary opacities (e.g. ground glass opacities or consolidation). Suboptimal images were defined as films wherein the patient had inadequate inspiration or excessive rotation, and consensus was obtained by all three radiologists before labelling an image as suboptimal.

Categorical variables were compared using Fisher’s exact test and continuous variables were compared using Mann-Whitney U test. All statistical tests were two-tailed, and P-value <0.05 was considered significant. A multi-variate logistic regression model was constructed imputing age, gender, comorbidities, and high-risk baseline CXR to identify factors independently associated with severe illness. All statistical analyses were performed using Stata Release 13 (StataCorp, College Station, TX, USA).

## Results

120 patients were admitted and isolated during the study period. 11 patients were excluded due to incomplete data (n = 5), negative nasopharyngeal swabs (n = 4), suboptimal initial radiograph due to rotation (n = 1) and no radiographs taken (n = 1). Of the remaining 109, median age was 42.5 years (interquartile range [IQR] 32–56), 58 (53.2%) were male, and 30 (27.5%) had comorbidities. Median day of presentation was day 4 of symptoms (IQR 1–8). Symptoms at presentation in order of frequency were fever (72.4%), cough (68.8%,) sore throat (44.0%), sputum production (29.4%), diarrhea (17.4%), and dyspnea (13.8%). Two patients were asymptomatic.

7 patients (6.4%) required supplemental oxygen at presentation in view of hypoxia. 19 patients (17.4%) went on to require supplemental oxygen during the course of admission, and 11 (10.1%) required ICU admission. Median day of illness of first requirement of supplemental oxygen was day 7 of symptoms (IQR 3–8). As the study was conducted in a period was before any proven COVID-19 treatment was identified, patients received a variety of pharmacologic interventions including lopinavir-ritonavir, hydroxychloroquine, interferon-beta, and remdesivir. Interventions were administered on an off-label use, apart from remdesivir which was administered as part of ongoing clinical trials during the study period.

**Fig 1:**
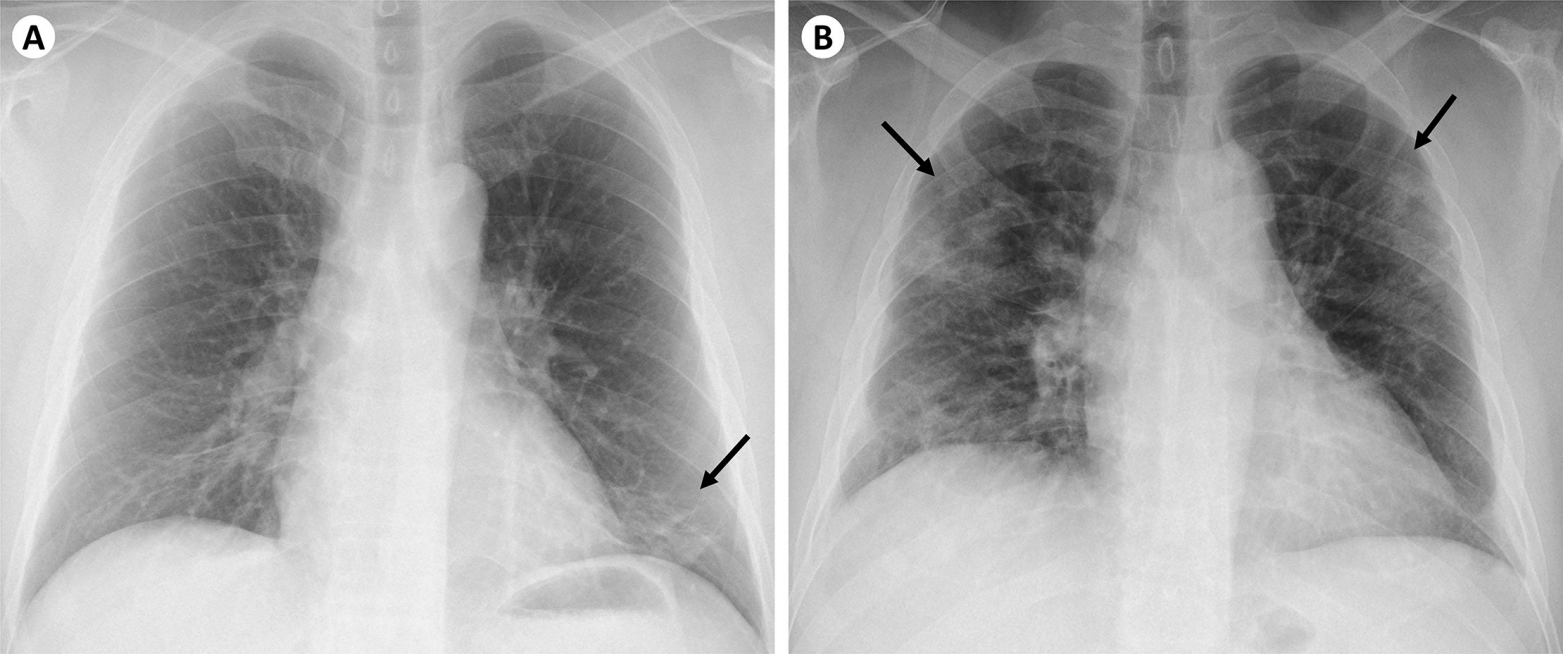
Example radiographs of (A) low-risk CXR and (B) high-risk CXR. (A) Low-risk CXR showing unilateral opacity (demarcated by black arrow) in the left lower zone. This patient’s symptoms resolved after 3 days and he did not require supplemental oxygen throughout his admission. (B) High-risk CXR showing bilateral, multifocal opacities involving the upper zones (demarcated by black arrows). This patient developed hypoxia requiring supplemental oxygen during his admission.

In the initial analysis of 40 patients (36.7%) with abnormal baseline CXR, presence of bilateral opacities, multifocal (in more than one zone) opacities, or any upper or middle zone opacity were significantly associated with supplemental oxygen requirement (Table 1). These features were defined as high-risk CXR features, and a high-risk CXR defined as having at least one of these high-risk features. Examples of low-risk CXR and high-risk CXR are illustrated in Fig 1. We then compared patients with high-risk CXR at baseline to patients without baseline high-risk CXR. There was no significant association between predominant zone of opacity with disease severity.

**Table 1:** Analysis of subset of 40 patients with initial abnormal CXR to identify radiographic features associated with supplemental O2 requirement.

	Supplemental O2 (n = 15) (%)	No supplemental O2 (n = 25) (%)	Odds ratio (95% CI)	*P*-value
**Radiographic feature**				
Bilateral opacities	14 (93.3)	10 (40)	21.00 (2.37–185.93)	**0.001**
Multifocal opacities (>1 zone)	14 (93.3)	13 (52)	12.92 (1.47–113.77)	**0.013**
UZ predominance	0 (0)	0 (0)	NA	NA
MZ predominance	1 (6.7)	3 (12)	0.52 (0.05–5.55)	0.586
LZ predominance	12 (80)	18 (72)	1.56 (0.33–7.24)	0.715
Any UZ/MZ opacity	13 (86.7)	13 (52)	5.99 (1.11–32.26)	**0.040**

O2 = oxygen; CI = confidence interval; UZ = upper zone; MZ = middle zone; LZ = lower zone; NA = not applicable

Of the entire cohort, 29 patients (26.6%) had a CXR with at least one high-risk feature at presentation. This group of patients had significantly greater odds of requiring supplemental oxygen (odds ratio [OR] 14.0, 95% confidence interval [CI] 3.90–55.60) and mechanical ventilation (OR 17.55, 95% CI 3.17–173.30) (Table 2). They were also older and had greater frequency of comorbidities. High-risk CXR was associated with significantly lower absolute lymphocyte count and higher C-reactive protein (CRP) and lactate dehydrogenase (LDH) levels, which are known laboratory markers associated with disease severity [13]. Patients with high-risk CXR were more likely to present with dyspnea and less likely to present with sore throat; reflecting the primarily pneumonic disease manifestation as opposed to a limited upper respiratory tract infection. There were no significant differences in the other presenting symptoms (including symptom duration) or investigations analyzed.

**Table 2:** Baseline characteristics and clinical outcomes of patients with initial low-risk CXR vs high-risk CXR.

	Low-risk CXR (n = 80)	High-risk CXR (n = 29)	Odds ratio (95% CI)	P-value
**Demographics**				
Age	37 (30–51.5)	55 (44–62)		**<0.001**
Male sex	42 (52.5)	16 (55.2)		0.831
Comorbidities	16 (20.0)	14 (48.3)		**0.004**
**Symptoms at presentation**				
Duration of symptoms (days)	4 (1–8)	5 (2–8)		0.254
Fever	57 (71.3)	22 (75.9)		0.809
Cough	52 (65.0)	23 (79.3)		0.170
Sputum production	22 (27.5)	10 (34.5)		0.485
Dyspnea	6 (7.5)	9 (31.0)		**0.003**
Sore throat	44 (55.0)	4 (13.8)		**<0.001**
Diarrhea	13 (16.3)	6 (20.7)		0.579
**Baseline investigations**				
White blood cell count (x10^9^/L)	4.70 (4.00–5.90)	4.60 (3.96–5.90)		0.817
Platelet count (x10^9^/L)	200 (177–253)	178 (140–265)		0.303
Neutrophil count (x10^9^/L)	2.72 (2.05–3.72)	3.32 (2.21–4.46)		0.225
Lymphocyte count (x10^9^/L)	1.26 (0.91–1.64)	0.91 (0.68–1.31)		**0.004**
Creatinine (umol/L)	66 (53.5–81)	67 (56–79)		0.665
C-reactive protein (mg/L)	4.7 (1.3–13.6)	50.6 (7.1–108.3)		**<0.001**
Lactate dehydrogenase (U/L)	395 (359–470)	538 (447–721)		**<0.001**
**Clinical outcomes**				
Supplemental O2 requirement	5 (6.3)	14 (48.3)	14.00 (3.90–55.60)	**<0.001**
ICU admission	2 (2.5)	9 (31.0)	17.55 (3.17–173.30)	**<0.001**

CXR = chest radiograph; CI = confidence intervals; O2 = oxygen; ICU = intensive care unit

High-risk CXR is defined as having any one of the following three criteria: (i) bilateral opacities, (ii) multifocal opacities, or (iii) presence of upper zone or middle zone opacity.

Categorical variables are expressed as number (percentage), and continuous variables are expressed as median (interquartile range).

In the multivariable logistic regression analysis which included age, gender, and presence of comorbidities, high-risk baseline CXR was independently associated with requirement of supplemental oxygen (adjusted odds ratio 8.38, 95% CI 2.43–28.97, *P* = 0.001). The other variables in the multivariable model did not meet statistical significance in association with supplemental oxygen requirement.

## Discussion

While a majority of COVID-19 patients have mild illness, five to ten percent of patients presenting to hospital develop severe illness requiring higher acuity of care [14]. Recognition of features associated with disease severity is important to prioritize limited healthcare resources for sicker patients, especially if the pandemic progresses unabated, placing greater pressure on healthcare systems.

We identified high-risk CXR features that were associated with severe illness, as defined by hypoxia requiring supplemental oxygen. Having a high-risk CXR with at least one of these features was also significantly associated with known laboratory markers elevated in severe COVID-19 [13]. Although these simplified criteria for high-risk CXR are not as precise as quantitative CXR scoring techniques, and are thus not comparable in terms of sensitivity in predicting severe illness; these features can be easily recognized by healthcare workers with limited radiology training, and can thus be readily implemented into routine assessment in the primary care or ambulatory setting. Furthermore, performing and reading a CXR has a rapid turnaround time, faster than laboratory investigations, allowing for swifter recognition of a patient with potentially severe illness.

Other studies have assessed clinical risk scoring tools utilizing varying combinations of clinical and laboratory parameters to predict risk of developing severe illness [15–17]. Two risk scores by Galloway et al and Liang et al incorporated a qualitative CXR score and a categorial parameter (presence of absence of chest radiograph abnormality) respectively in their risk scores [18, 19]. The qualitative high-risk CXR criteria that we have identified in this study could be incorporated into future risk scores for further refinement, providing the same benefit over quantitative radiographic indices in terms of ease of implementation.

Of note, the association of upper or middle zone opacities with severe illness has not been previously emphasized. The typical radiographic appearance of pulmonary involvement in COVID-19 is that of lower zone opacification [20]. Upper or middle zone involvement may thus signify more extensive pulmonary disease, explaining the associated poorer clinical outcomes. This is an easily recognizable CXR feature that should be highlighted to clinicians conducting initial assessment of suspected COVID-19 patients.

Our study is limited by its small sample size, and may thus have missed smaller but clinically relevant differences in outcomes. Secondly, image acquisition was not standardized as this study had to be pragmatically incorporated into the workflow of managing a high volume of patients during the outbreak. Variation in CXR images between PA and AP films could have affected the interpretation of the results, though we assess this impact to be minimal. Thirdly, CXR interpretation was conducted by experienced radiologists in this study. Although we intentionally kept the CXR interpretation as simple as possible, further study should explore the utility and applicability of this categorization by general medical practitioners.

We also recognize the limitations of CXR as a predictive tool for severe disease, which may only manifest in the second week of illness [14]. CXRs taken within the first week may thus be unable to predict subsequent progression, and a normal of low-risk CXR early in the illness course may not identify a potentially very ill patient. Attention to close follow-up should thus be made for patients with a normal baseline CXR, but with other risk factors for clinical deterioration. Nevertheless, as CXR is cheap and portable, recognition of these specific high-risk CXR features would greatly benefit timely referral from community-based to acute care facilities. Community-based healthcare facilities managing COVID-19 patients should also ensure that there is readily-accessible radiography to guide triage and patient disposition.

## Conclusion

In conclusion, we identified several high-risk CXR features associated with severe COVID-19 requiring supplemental oxygen, and used this to formulate criteria to define a baseline high-risk CXR. This can be utilized for assessment and triage of patients in the ambulatory setting, or incorporated into other multi-variable risk assessment scores.

## Supporting information

S1 Data (XLSX)
